# Neutrophil lymphocyte ratio and heart type fatty acid binding protein as a prognostic marker in Myocardial infarction within 48 h of admission

**DOI:** 10.1186/s43044-024-00489-z

**Published:** 2024-05-21

**Authors:** K. G. Nikhil, K. T. Jayakumar, P. J. Shiny, N. K. Ramya, J. S. Kumar

**Affiliations:** 1 Department of General Medicine, SRM Medical College Hospital and Research Centre, SRMIST, Kattankulathur, Kancheepuram District, Tamil Nadu 603209 India; 2 Department of Medical Research, SRM Medical College Hospital and Research Centre, SRMIST, Kattankulathur, Tamil Nadu 603209 India

**Keywords:** STEMI, Neutrophil–lymphocyte ratio, H-FAB, Acute coronary syndrome, Mortality

## Abstract

**Background:**

The neutrophil to lymphocyte ratio (NLR) is a measure of systemic inflammation, whereas Heart type fatty acid protein (HFABP) is a cytosolic protein released early after acute coronary syndrome (ACS). The aim of this research study is to determine whether NLR and H-FAB are useful in predicting the prognosis in patients with ST segment elevation myocardial infarction (STEMI) 48 h after admission. This is a prospective observational study conducted on 97 patients who had been admitted to emergency room with ST-elevation myocardial infarction in their ECG in a tertiary care centre of south India. The neutrophil–lymphocyte ratio was measured at the time of admission, 24th hour and 48th hour, and then compared with the outcome. To determine their significance in the MI episode, troponin-I and H-FABP were also measured.

**Results:**

A significant correlation was found in the final outcomes of patients and the NLR at the time of admission and at 48 h (*p* = 0.01). Additionally, a substantial correlation between NLR and various degrees of LV dysfunction was also observed (*p* = 0.01). H-FABP was found to be positive in all 97 of the patients examined, whereas Troponin-I was only found to be positive in 56.7%.

**Conclusion:**

The study's findings, indicated strong correlations between NLR and LVEF, indicated that NLR might serve as an early predictor of cardiac events which could be either poor prognosis or higher mortality. This research found that H-FABP may serve as an early MI diagnostic marker.

## Background

The main cause of death and morbidity in middle-aged and older people is coronary artery disease. Acute coronary syndrome is a term used to describe patients with unstable angina, non-ST-elevation myocardial infarction, or ST-elevation myocardial infarction (ACS) [1]. A thorough risk assessment of these patients would help medical professionals to determine the prognosis, which aids in guiding the treatment plan and give the patient excellent information [2].A risk prediction prototype must be simple that can use diagnostic health risks that are easily accessible at healthcare facilities like the emergency room in order to be applied in clinical practise.

The neutrophil-to-lymphocyte ratio (NLR), which has been related to negative outcomes in people with acute coronary syndromes (ACS) and established coronary heart disease, has emerged as a key inflammatory marker for categorising cardiovascular risk [3]. Elevated NLR is individually and strongly associated with a higher risk of complications and death in post-MI patient populations, according to the data as reported by White et al., 2019 [4]. An index study conducted during June 2021to May 2022 also indicated that the final outcome of STEMI patients could be predicted using NLR that also correlated with poor LV function [5]. Patients with ACS are more likely to pass away, especially in the first 30 days. It has been shown that NLR can predict minimal in-hospital illness and mortality. Additionally, earlier research has connected the NLR to cardiovascular death in STEMI patients as well as major adverse cardiac events (MACE) that occur in hospitals [6].

The FABP family, which includes cardiac FABP (H-FABP), also known as breast-derived growth inhibitor, is found in tissues with a high demand for fatty acids, such as the heart, skeletal muscle, brain, kidney, adrenal gland, mammary tissue, and blastocysts. In striated muscle cells, H-FABP is widely distributed in the cytoplasm and is rapidly released in reaction to cardiac injury [7]. The significance of H-FAB as a prognostic FABP in patients with unstable heart disease is limited. Hence, studies testing the value of HFABP in early diagnosis of acute coronary syndrome in patients who have been admitted for ST-elevation MI within 48 h for MI management are scarcely reported and are scarcely reported [8]. These studies individually represent the NLR and H-FAB correlations with the cardiovascular events in STEMI ACS. Therefore, the current study evaluated the correlations of NLR and H-FAB with the cardiovascular patients individually.

## Methods

This is a prospective observational study involving 97 study participants who had been admitted to Intermediate Care Unit or Intensive Coronary Care Unit with angina lasting for more than 30 min and ST-elevation for MI management in SRM Medical College Hospital and Research Centre, Chennai, India. The research was conducted with consent from the patient and the approval of the Institutional Ethics Committee (IEC). The sample size was estimated to be 100 based on the prevalence of angina with the sampling frame as the angina presentation for more than 30 min along with the ST-elevation (greater than 1 mm in limb leads or higher than 2 mm in a minimum of 2 contiguous chest leads) without diabetes mellitus, and getting admitted within 48 h of angina onset for myocardial infarct ion management along with continuous monitoring for any major adverse cardiac events in IMCU or ICCU. Purposive sampling was used in the research, and 97 eligible STEMI patients who were admitted during the study period were included. The most crucial inclusion criteria were patients with non-diabetic STEMI who were admitted within 48 h of the onset of angina or symptoms, who had undergone blood tests including albumin, echocardiography, and angiography.

**Fig. 1:**
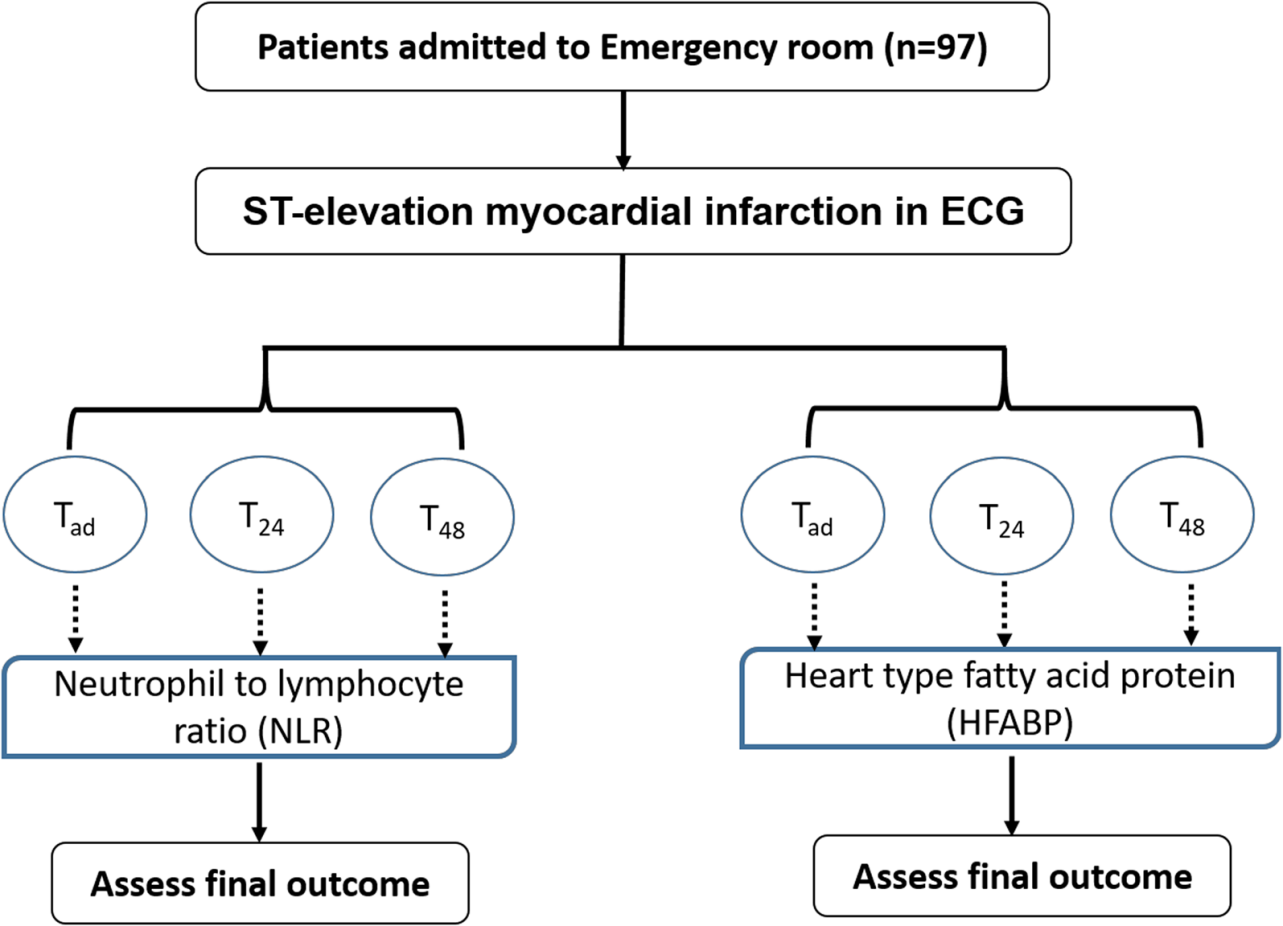
The overall representation of the study

The study participants underwent physical examination, routine blood tests like blood glucose, lipid profiles and troponin I, as well as tests specific to the study, like neutrophil and lymphocyte counts to determine the neutrophil–lymphocyte ratio and H-FABP. They also underwent 12-lead ECGs to confirm ST-elevation and evaluate the condition of their cardiac function. If the patient was hemodynamically stable, family members or the patient themselves were consulted for the patient's prior medical background. Patient information includes demographics like age and gender, past medical history like smoking, alcohol use, cholesterol, hypertension, neutrophil–lymphocyte ratio at the time of admission, H-FABP at the time of admission, changes in the ST segment of the ECG, and echocardiography for the presence of arrhythmias or other cardiac rhythmic changes, among other things. From the time of admission until they were discharged from the appropriate facility, along with their outcome, every patient who had been admitted with STEMI and managed for MI underwent continuous hemodynamic monitoring. The representation of the study is described in Fig. 1.

### Estimation of neutrophil -lymphocyte ratio

A volume of three millilitres of blood was collected from patients in containers with EDTA Ethylene Di-amine Tetra-acetic Acid (EDTA). The sample was subjected to a complete blood count for both differential and total count using an automated coulter counter and a manual investigation. The obtained neutrophil count and lymphocyte counter were recorded. The neutrophil lymphocyte ratio was calculated using the below formula:

$${\text{Neutrophil-Lymphocyte\;ratio}} = \frac{{{\text{Neutrophil\;count}}}}{{{\text{Lymphocyte\;count}}}}$$

### Detection of heart type-fatty acid binding protein

The heart type- fatty acid binding protein Kit for detecting heart attacks which was procured from Randox Laboratories, Ltd, UK. It is a quality assay aimed for screening the coronary reperfusion injury. The kit identified the individuals to be positive or negative for MI. The procedures was performed according to the standard norms or protocols as mentioned in the instruction manual of the kit. Based on the interpretation, the patients were classified as positive and negative. The percentage of increase in NLR from admission to the respective time in hours was calculated using the below formula:

$$\begin{aligned} & {\text{NLR\;percentage\;increase\;from\;admission\;to\;time}}\;\left( h \right) \\ & \quad = \frac{{\left( {{\text{NLR\;at\;time }}\left( {{\text{hour}}} \right){ } - {\text{at\;admission}}} \right){ }}}{{{\text{NLR\;at\;admission}}}} \times 100 \\ \end{aligned}$$

All the study-specific parameters were prospectively recorded and statistical analysis using SPSS version 25 was performed. A *p*-value of less than 0.05 was regarded as statistically significant when the statistics were run at a 95% confidence level.

## Results

Out of the 97 patients, there were 36 females and 61 males with a mean age of 66.1 years. The youngest patient to suffer STEMI in our study was 35 years old, while the oldest was 92 years old. The mean neutrophil–lymphocyte ratio was found to be the highest during 48th hours (5.8 ± 3.1), than 24th hour (4.0 ± 2.7) and at admission (3.9 ± 2.1) (Table 1).

**Table 1:** NLR values at different times

Parameter	Mean ± SD (range)
NLR at Admission	3.9 ± 2.1 (1.3–11.6)
NLR at 24 h	4.0 ± 2.7 (1.1–12.7)
NLR at 48 h	5.8 ± 3.1 (1.6–12.7)

Among the 97 patients evaluated for STEMI ACS, all the patients were identified to be positive for H-FABP in the analysis. Troponin I was positive in 55.7% of the patients, and negative in 42.3% of them (Table 2).

**Table 2:** HFAB and troponin in the study subjects

Parameter	Result	N (%)
Heart type-fatty acid binding protein	Positive	97 (100%)
	Negative	0 (0.0%)
Troponin I result	Positive	55 (55.7%) 56.7
	Negative	42 (42.3%)–43.2%

The neutrophil–lymphocyte ratio has been distributed among the study participants according to their gender, age, smoking, alcohol habits, and also their health conditions like dyslipidemia and hypertension. Age was known to be associated significantly (*p* < 0.05) with the NLR at 48 h which was observed to be elevated in those above 66 years (6.1). Smokers, alcoholics, and hypertensive patients also showed an elevated NLR at admission and 24 h, but this association was not significant statistically (*p* > 0.05). High triglycerides was found to be a significant risk factor in the elevated NLR values at admission, with a *p* value of 0.01 (Table 3).

**Table 3:** Distribution of variables with the NLR at different times

S. no.	Variable	Classification	NLR			*p* value
			At admission	24 h	48 h	
1	Gender	Male (n = 61)	3.8 ± 2.2	4.1 ± 2.8	5.6 ± 3.0	0.73
		Female (n = 36)	4.0 ± 1.8	3.9 ± 2.6	6.1 ± 3.2	
2	Age classification	< 66 years (n = 44)	4.3 ± 2.3	4.3 ± 2.8	5.4 ± 3.0	0.005
		> 66 years (n = 53)	3.5 ± 1.8	3.8 ± 2.6	6.1 ± 3.1	
3	Smoking	Smokers (n = 48)	4.0 ± 2.1	4.2 ± 2.8	5.7 ± 2.9	0.47
		Non-smokers (n = 49)	3.7 ± 2.1	3.8 ± 2.6	5.9 ± 3.3	
4	Alcohol	Alcoholic (n = 48)	4.0 ± 2.1	4.2 ± 2.8	5.7 ± 2.9	0.72
		Non-alcoholic (n = 49)	3.7 ± 2.1	3.8 ± 2.6	5.9 ± 3.3	
5	Hypertension	Present (n = 71)	3.8 ± 2.0	3.9 ± 2.6	5.8 ± 2.9	0.415
		Absent (n = 26)	4.0 ± 2.4	4.4 ± 3.0	5.6 ± 3.4	
6	Triglycerides	High (n = 41)	4.5 ± 1.9	3.9 ± 2.6	5.6 ± 3.0	0.01
		Normal (n = 56)	3.4 ± 1.9	4.2 ± 2.8	5.9 ± 3.1	

The neutrophil–lymphocyte ratio was compared with the outcome of patients in the study (Table 4), and we found that the NLR at 48 h has shown a significant association (*p* = 0.00), while the NLR at admission and 24 h has no change with the outcome. Elevated NLR at 48 h has predicted a worst prognosis in the STEMI patients (NLR at 48 h = 11.8 ± 0.78).

**Table 4:** Outcome of STEMI patients with NLR

Outcome	NLR (mean ± SD)		
	Admission	24th hour	48th hour
Alive	3.9 ± 1.9	3.9 ± 2.5	5.1 ± 2.4
Dead	3.4 ± 3.0	5.3 ± 4.0	11.8 ± 0.78
*p* value	0.59	0.29	0.000*

Elevated NLR at the time of admission was also able to determine the outcome in terms of ejection fraction levels showing mild and severe LV dysfunction which was significant. The study analysis lucidly defines that the percentage of increase in NLR is critical between 24 to 48th hours where there is a sharp elevation in the concentration of NLR as revealed than when compared for the percentage increase in NLR between admission and 24th hour. The highest percentage of increase in NLR concentration was observed at 48 h from the time of admission followed by an increase in NLR levels at 24 h. The NLR values were more specific between 24 and 48 h than 24 h from admission and statistically significant (*p* = 0.001*).

## Discussion

Only 55 of the participants (56.7%) were found to be positive for troponin-I at the time of admission, and the remaining 42 patients were reported to be positive within 24 h of admission, according to the results. A non-significant change in the NLR levels between the troponin positive and negative patients was observed. NLR was a indicatory factor for anticipating cardiovascular fatality occurrences, and people in the elevated NLR group had a greater likelihood of death from the disease, supporting the aforementioned viewpoint in similar to Lin et al. [9].

Lymphocytes play a pathophysiological role in immune system regulation, while neutrophils and monocytes play a key role in the inflammatory reaction. NLR may be more clinically predictive of the development of illness than a single white blood cell count [10]. Neutrophil-to-lymphocyte ratio (NLR) has been studied by Ji et al. (2021) in relation to predicting the short-term outcome of NSTEMI and STEMI. After adjusting for factors like age, sex, diabetes history, smoking history, and high LDL-C, the logistic regression showed that patients with NLR > 5.509 had a greater hazard risk of death than patients with NLR less than five [11].

The average concentration of NLR with different grades and types of LV ejection fraction and arrhythmias. LV ejection fraction showed excellent correlation. Fraser et al., observed in his study that Patients with NLR in the highest tertile had significantly worse outcome than those in the lowest independent of LVEF (< 40%: HR 2.75; LVEF ≥ 40%: HR 1.51) [12]. In a study on patients with peritoneal dialysis revealed that the elevated NLR was also associated with the all-cause mortality rendering the importance of NLR monitoring to predict outcomes [13]. Another study reports that elevated NLR indicated adverse outcomes of heart failure and worse outcomes in cardiovascular events indicating the potential of NLR as an economical biomarker [14].

There was no significance to the cardiac events of arrhythmia in our study and no other significant cardiac occurred with our study patients during the investigation. Banu et al. [15] tested on 66 patients whether H-FABP could be served as biomarker for the prediction of ACS alone or in combination. In this study, only 15.2% of patients had positive H-FAPB values whereas in our study, all the patients were found to be positive for H-FAB during the initial time of admission itself. The sensitivity, specificity, PPV, and NPV values of all commonly used biomarkers for the diagnosis of ACS increased when H-FABP was added.

Another study found that using the H-FABP alone or in combination with commonly used biomarkers had no discernible effect on the early diagnosis and exclusion of ACS unlike our study [16]. Another study reports the effectiveness of H-FAB to be used to predict the severity of CAD [17]. The sensitivity of HFABP and its specificity for the early diagnosis of ACS were found to be between 39–78.5% and 78.2–94% in various studies, but the present investigation had not assessed the sensitivity and specificity of the investigations but our study had depicted that H-FAB is found to be better through correlation analysis [18]. The limitations of the study is that it was conducted only in a single centre and the duration of the study is for a shorter time period. The study could draw firm conclusions if the sample size would be larger in a multi-centric study.

## Conclusions

NLR was able to predict the prognosis of STEMI patients, especially 48 h after the admission, and moreover, it also aids in the assessment of the functional status of LV function. It was also understood that H-FAB could be utilized as biomarker in STEMI ACS non-diabetic individuals for detecting MI. Future studies should be performed in larger cohorts of population in detecting the sensitivity and specificity of NLR for predicting MI.

## Data Availability

The datasets used and/or analysed during the current study is available from the corresponding author on reasonable request.

## Abbreviations

NLR: Neutrophil to lymphocyte ratio
HFABP: Heart type fatty acid protein
STEMI: ST segment elevation myocardial infarction
ACS: Acute coronary syndrome
MACE: Major adverse cardiac events
MI: Myocardial Infarction
